# Supplementary material for: Splice-Junction-Based Mapping of Alternative Isoforms in the Human Proteome
Source: Cell Rep. Author manuscript; Available in PMC 2020 Jan 15. (PMC6961840; doi:10.1016/j.celrep.2019.11.026)
Supplement: 2 [file NIHMS1546469-supplement-2.zip › DF1/PXD006675/RightAtrium/RightAtrium_17_TTN_RVEAEPAEVTEFR.pdf]

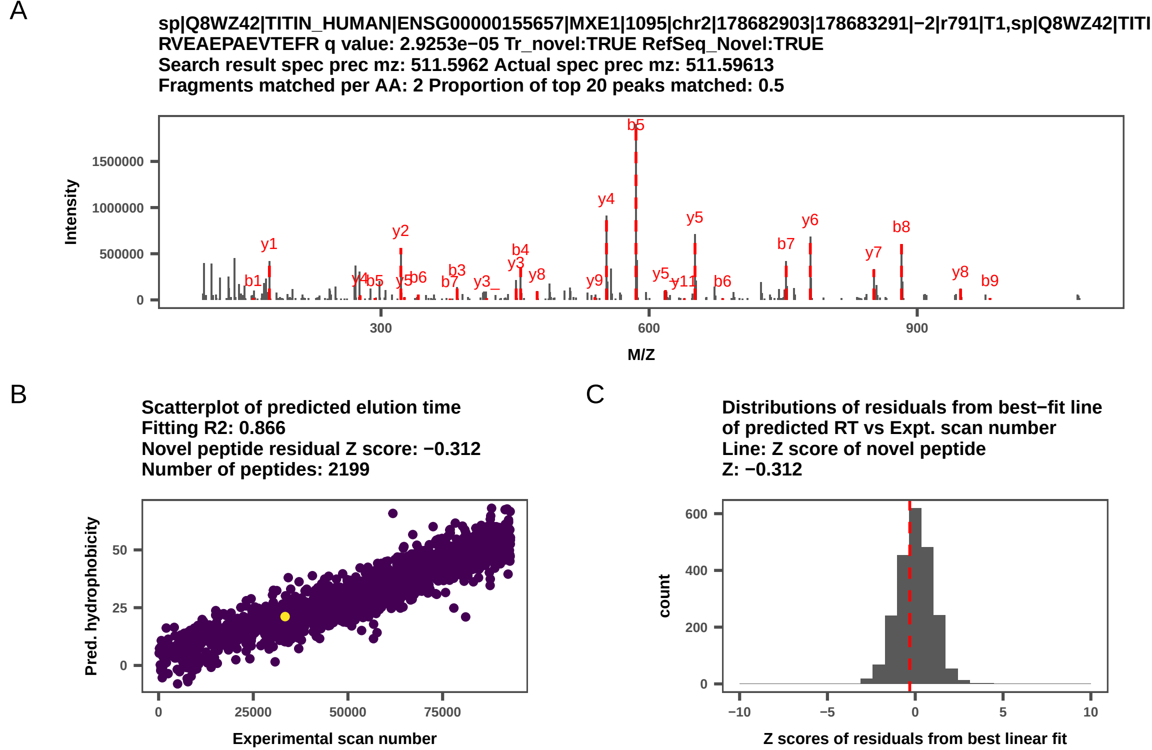
A
sp|Q8WZ42|TITIN_HUMAN|ENSG00000155657|MXE1|1095|chr2|178682903|178683291|−2|r791|T1,sp|Q8WZ42|TITI
RVEAEPAEVTEFR q value: 2.9253e−05 Tr_novel:TRUE RefSeq_Novel:TRUE
Search result spec prec mz: 511.5962 Actual spec prec mz: 511.59613
Fragments matched per AA: 2 Proportion of top 20 peaks matched: 0.5
Intensity
1500000
1000000
500000
0
300
600
900
M/Z
b5
y4
y5
y6
b8
y2
y1
b7
y7
b4
y3
b3
y8
b1
y4
b5
y5
b6
b7
y3_
y9
y5
y11
b6
y8
b9
B
Scatterplot of predicted elution time
Fitting R2: 0.866
Novel peptide residual Z score: −0.312
Number of peptides: 2199
Pred. hydrophobicity
50
25
0
0
25000
50000
75000
Experimental scan number
C
Distributions of residuals from best−fit line
of predicted RT vs Expt. scan number
Line: Z score of novel peptide
Z: −0.312
count
600
400
200
0
−10
−5
0
5
10
Z scores of residuals from best linear fit
